# Save the microbes to save the planet. A call to action of the International Union of the Microbiological Societies (IUMS)

**DOI:** 10.1186/s42522-023-00077-2

**Published:** 2023-03-06

**Authors:** Rino Rappuoli, Paul Young, Eliora Ron, Simone Pecetta, Mariagrazia Pizza

**Affiliations:** 1 Monoclonal Antibody Discovery (MAD) Lab, Fondazione Toscana Life Sciences, Siena, Italy; 2 Present address: Fondazione Biotecnopolo di Siena, Siena, Italy; 3 University of Queensland, QLD, St Lucia, Australia; 4 Tel Aviv University, Ramat Aviv, Tel Aviv, Israel; 5 Research and Development Center, GSK, Siena, Italy; 6 Present address: Moderna Inc., Cambridge, MA USA; 7 Present address: Imperial College South Kensington Campus, London, UK

**Keywords:** Microbes, Biodiversity, Climate change, Antimicrobial resistance (AMR), Antibiotics, Planetary health

## Abstract

Our planet is populated by at least a trillion species of microorganisms. Every life form is sustained by them and they make the planet habitable. Only a minority of them, about 1400 species, cause infectious diseases that are responsible for human morbidity, mortality, pandemics and the resulting huge economic losses. Modern human activities, environmental changes and the attempt to control infectious agents using broad spectrum antibiotics and disinfectants jeopardize the global microbial diversity. The International Union of the Microbiological Societies (IUMS) is launching a call to action to mobilize all microbiological societies globally to promote the development of sustainable solutions to control infectious agents while preserving the global microbial diversity and the healthy life of our planet.

## Introduction

Over recent decades, an increased awareness of the human impact on planet Earth has begun to mobilize populations around the world. Clearly our generation is taking from our planet more resources than it can sustainably deliver, and this has an impact on climate, biodiversity and ecosystems which in turn impact human health and wellbeing. We are in search of solutions that could decrease carbon dioxide emissions, containing a global temperature rise to below 2 C° by 2100 and so limiting the impacts of climate change [1]. We are also beginning to recognize the impact of human activities on living organisms and biodiversity, with 705 vertebrate species and 571 plant species having already been pushed to extinction in the past five centuries. A further one million species of animals and plants are currently threatened with extinction, including large terrestrial animals, marine mammals, animal pollinators, marine corals, and fish stocks [2]. Moreover, increased consumption of limited resources and intensified industrial processes are degrading ecosystems and biodiversity with declining indicators of the status of Nature, leading to a deterioration of the fabric of life itself [2]. However, we are not yet paying attention to the consequences of human impact on microorganisms. This impact on microbial species is happening quietly, below the radar of most scientists, politicians, and the public, but it should not be underestimated because microorganisms are at the origin of life and sustain all life forms.

## Microbial diversity is essential for life on our planet

Microorganisms were the first form of life to appear on our planet 3.8 billion years ago. Two and half billion years ago they were instrumental in the emergence of the first eukaryotic cell from which all animals and plants, including humans, were derived. About forty trillion (﻿10^12^) microbes live in the gut of every human, with an increasing number of studies highlighting the key role they play in our physiology: from providing essential metabolic functions to influencing our own immune responses. Indeed, they play important roles in the survival and functioning of every form of life. Today our planet is populated by about a trillion species of microorganisms [3]. They include bacteria - the vast majority – but also viruses, fungi, and unicellular microorganisms. At any one time there are roughly five million trillion trillion (10^30^) living bacteria and 10^31^ phages (viruses that infect bacteria) that attack them, killing 40% of them every day [4–6]. Microorganisms are the essence of life, and the driving force of evolution. They evolve at a speed that would require 10,000 years of experiments in the laboratory just to match 1 day of natural evolution [7]. High biodiversity and fluctuation of microbial communities are critically important for species interaction and function [8, 9]. A minority of them (1415 species, 217 viruses or prions, 538 bacteria, 307 fungi, 66 protozoa, and 287 helminths) are also responsible for causing infectious disease with few having shaped human history and its evolution over time by killing hundreds of millions of people [10, 11]. The remaining 999 billion species of microorganisms are useful and essential to the life on our planet. They are present in the water, soil, air, in harsh environments such as deep-sea vents and hot springs with boiling water, in obscure caves where they get their nutrients from sulfur, and they can resist radiation better than any other living organism. We rely on them to make food (bread, wine, beer), to treat wastes (sewage treatment plants), and to make essential medicines such as antibiotics [12]. During the 3.8 billion years on our planet, they evolved genes that can perform many of the functions we depend on. They can make chemical reactions and material transformations at room temperature that we can only perform at extremely high temperatures with heavily polluting industrial plants. We engineer them in our laboratories to produce biological molecules, drugs, and enzymes.﻿ The application of microbes can also help in finding sustainable solutions to most of the critical problems that face our planet such as climate change, environmental degradation, health and energy needs, provided that we learn how to use them wisely and we stop destroying them either inadvertently or by our ambition to sterilize the environment that surrounds us. In summary, life on our planet is fully dependent on microbes and the almost limitless functions performed by them. Microbial diversity is essential to maintaining the functions that support life on the planet and therefore it is important to monitor and preserve that diversity. Below are some of the challenges facing the microbial world today and possible ways to overcome them.

## Useful microbes

Microbes are present everywhere, and they make the planet habitable. The oceans probably carry a large part of the microbial mass of the planet. Indeed, microbes represent from 50 to 90% of the biomass of the oceans. The number of microbial cells in the waters of the planet has been calculated to be in the range of 10^30^ with a mass equal to the weight of 240 billion African elephants [13]. In 2004, Craig Venter identified 1800 genomic species and 148 previously unknown bacterial phylotypes by sequencing the genome of microorganisms collected by filtering 200 l of water from the Sargasso sea [14]. On dry land, one gram of surface soil can contain more than 10^9^ bacterial and archaeal cells, trillions of viruses, tens of thousands of protists and 200 m of fungal hyphae [15]. Microorganisms colonize all living organisms and, as previously mentioned, about 40 trillion microbes reside in the gut of every human [16]. Since we estimate that the number of human cells that make up our body are around 30 trillion, this means that we are as much microbes as we are human [16]. Their presence, in addition to providing vitamins, helps the digestion by breaking down fibers and starch, supports endocrine metabolism, and is essential for the formation and maintenance of our immune system. Mice born without exposure to microbes - germ free mice – have fewer and smaller Peyer’s patches and mesenteric lymph nodes and show extensive defects in the development of gut-associated lymphoid tissues and antibody production [17]. Immune dysfunctions are also observed when mice are treated with antibiotics early in life, revealing the key role of microbial colonization on immune maturation [18]. Recent studies have shown that the ability of our immune system to fight tumors, infections, and chronic inflammation is heavily dependent on the presence of a healthy microbiome [19, 20]. Interestingly, supplementation of microbes can also help restore a healthy microbiota, with clear therapeutic opportunities that are currently being pursued.

Microbes also support many industrial activities, starting from traditional fermentation of bread, cheese, beer, wine, to the production of chemicals, energy sources, enzymes, pharmaceuticals, and to waste treatment and pollution control where we use their ability to degrade virtually any product, including fossil oils and plastics [12]. Finally, we should not forget that microorganisms provide an important function in supporting agricultural productivity, by fixing atmospheric nitrogen and favoring the growth of high yielding crops, thus avoiding the pollution caused by use of synthetic nitrogen fertilizers.

## Microbes and infectious diseases

Human history has been shaped by the 1400 species of microorganisms that are responsible for infectious diseases. The recent pandemic caused by the SARS-CoV-2 virus, which estimates indicate has killed 20 million people so far and has had an impact of 28 trillion US dollars on the global economy, is a strong reminder of the impact they can have on health and society. Historical records are full of descriptions of infectious diseases that have changed human history. The deadliest plagues include the loss of 35% of the Athens population during the Peloponnesian War (430 B.C.), the Black Death that killed one third of the European population in 1347, and the infections imported by Europeans into the Americas, which decimated most of the native populations in the sixteenth century, essentially extinguishing whole civilisations. More recently, the 1918 pandemic influenza was responsible for the death of more than 40 million people [21]. Given this historical perspective, many have become more obsessive about hygiene, avoiding as much as possible any significant contact with microorganisms. To achieve this goal an array of tools has been developed that are designed to kill microbes and sterilize the environment with which we are in contact. However, most of these tools are not specific and the effort to get rid of the relatively small number of pathogens is jeopardizing the survival and the ecosystems of the one trillion species that sustain our life and our planet. Every day we clean houses and offices with chemicals that make the environment sterile and kill every microorganism. When we are sick, we use broad spectrum antibiotics that, in addition to the pathogen, kill all other bacteria within us, whether good or bad. We use hundreds of thousands of tons of antibiotics in agricultural farming every year, not with the aim of suppressing infectious disease but to accelerate the growth of chickens and cows. The chemicals and the antibiotics that we use locally are then disposed of in the environment and continue their effects on the waters and soils of the planet [22]. These approaches decrease microbial diversity and generate antibiotic resistant superbugs.

## Recommendations to preserve the planet’s microbial diversity

**Fig. 1:**
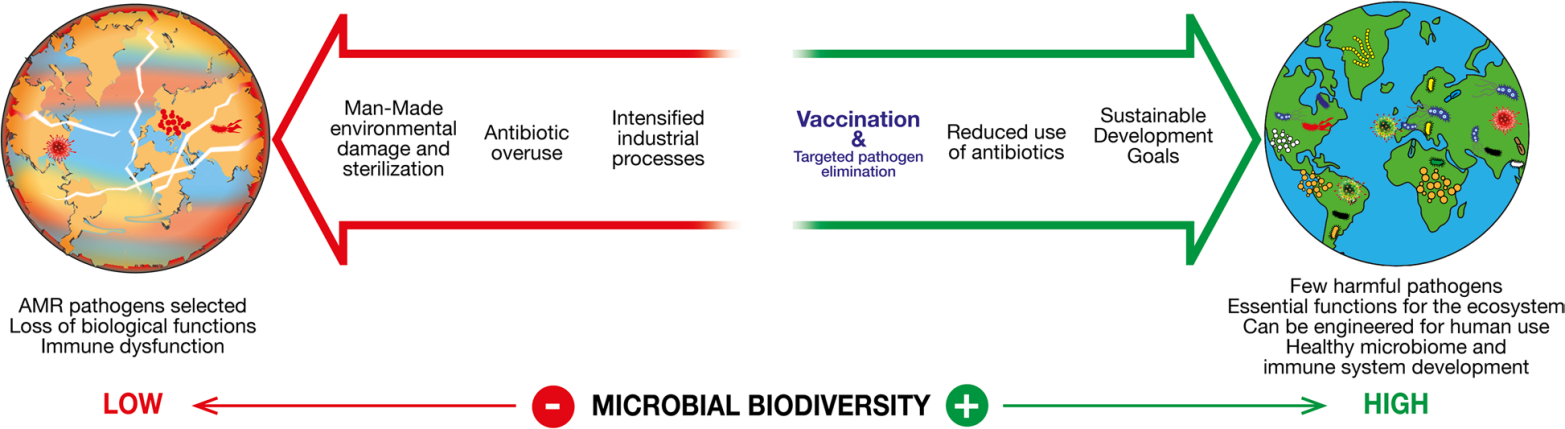
Competing challenges in the preservation of global microbial diversity

1. Target pathogens with sustainable solutions by minimizing the use of drugs and chemical agents with broad antimicrobial activity and invest in pathogen specific vaccines and pathogen specific treatments taking advantage of innovative technologies such as CRISPR-Cas and bacteriophages [23]. A﻿ sustainable approach to defend humans from the 1400 pathogenic species is vaccination (Fig. 1). It takes advantage of the natural property of our immune system that during the million years of evolution learned how to keep at bay microbial pathogens without impacting beneficial microorganisms. Vaccination is very specific, targets molecular epitopes that belong specifically to each pathogen and has limited, if any, bystander effect on other microorganisms. It is a natural way evolved by Nature to protect individuals from pathogens without impacting the environment and microbial diversity. It is the natural way by which mothers transfer their own specific immunity to their babies, before they can develop their own immunity. Moreover, vaccines can promote a One Health approach, protecting both people and animals from disease spread, in accordance with the WHO AMR Strategic Framework [24]. Finally, and along with more traditional vaccine approaches, we can take advantage of the new vaccine platform technologies, forged through the urgency of the recent pandemic, to accelerate development and implementation. Vaccines are an environmentally friendly, green solution for infectious diseases.
2. Reduce and possibly stop the use of antibiotics in animal feed. Antibiotics are routinely used as growth promoters and to prevent infections of animals, such as chickens, cattle, pigs, sheep and fish. It is estimated that, globally, 73% of all antibiotics are used in farm animals and less than 30% for humans [25]. Their use is increasing dramatically, with 63 thousand tons of antimicrobials used worldwide in 2010; it is expected that their use will increase to more than 100 thousand tons by 2030 [26]. The rising use of antibiotics is due to the increasing demand for food and the intensified livestock farming where large numbers of animals are grown in a very close environment with poor hygiene. While some countries in Europe have started to introduce regulations to reduce the use of antibiotics in animal feed, their use is increasing dramatically in China, India, Russia, Brazil and all low-income countries. Clearly, the use of 100 thousand tons of antibiotics every year not only promotes the evolution and dissemination of antibiotic resistant bacteria from animals to humans, but also contaminates the waters and soils of the planet driving the extinction of many useful microbes and the expansion of harmful clones of antibiotic resistant organisms [22, 27]. While the use of antibiotics in animal farming seems unstoppable, there are examples where this has been successfully done. For instance, in Norway, the use of antibiotics in aquaculture has been reduced by 90% through the introduction of vaccination in salmon farming [28]. The annual production of salmon has subsequently increased without the need to increase antibiotic supplements. This result is an example of what can be achieved if governments and industry work together to find sustainable solutions. Thus, One Health approaches aimed at (1) improving animal husbandry practices and housing conditions & hygiene, and (2) at promoting a better use of vaccination would counteract the increased use of antibiotics. Implementations of such solutions include animal population planning, veterinary surveillance for disease prevention and diagnosis, thorough cleaning of animal housing, human-animal spillover risk assessment, and quarantine periods for diseased animals. Such practices need particular attention in Low-Medium income countries where sustainability and profitability have also to be taken into consideration.
3. Support the achievements of the United Nations Sustainable Development Goals (SDGs). In 2015 the United Nations declared 17 Sustainable Development Goals (SDGs), with the ambition to promote prosperity and protect the planet’s health by 2030 [29]. While none of them addresses microbial diversity specifically, all of them have the aim of a healthy planet. This goal cannot be achieved without preserving the diversity of the global microbiome with some of the SDGs directly impacted by a healthy global microbiome. SDG 3 calls for good health and wellbeing and relies on the control of bad microbes using sustainable solutions such as vaccines. SDG 6 calls for clean water and sanitation relying on the action of microorganisms to adequately treat wastewaters. SDG 12 calls for responsible consumption and production relying in turn on the reduced abuse of antibiotics for farm animals, and the use of bacteria to fix nitrogen and reduce pollution caused by synthetic nitrogen fertilizers. Finally, SDG 14 (life below water) and SDG 15 (life on land) rely heavily on the diversity of their respective microbiomes which are essential to keep these environments healthy.

## Conclusions

There is no doubt that human activities are accelerating environmental changes, impacting the ecological communities, and jeopardizing the essential functions provided by the microbial species to our planet.

In this article we have identified a number of key overarching strategies whose broad implementation would help in preserving the planet’s microbial diversity:

- minimizing the widespread use of broadly acting antimicrobial agents alongside an increased focus on the development and application of targeted vaccine and therapeutic treatments that are more effective and sustainable,
- reducing the globally widespread overuse of antibiotics in agriculture which is helping drive the emergence of antibiotic resistant superbugs, and
- actively embracing the objectives of the UN Sustainable Development Goals in seeking and supporting solutions for a healthy planet.

The IUMS is deeply worried about the catastrophic consequences of current activities impacting the delicate balance of the microbial world and in turn the sustainability of healthy life. We are making a global call on all microbiological societies to unite and raise awareness of the importance of microbial diversity for the sustainability of our planet.

## Data Availability

Not applicable.

## Abbreviations

IUMS: International Union of the Microbiological Societies
SARS-CoV-2: Severe Acute Respiratory Syndrome Coronavirus 2
CRISPR-Cas: Clustered Regularly Interspaced Short Palindromic Repeats - CRISPR Associated System
WHO: World Health Organization
AMR: Antimicrobial Resistance
SDG: Sustainable Development Goals
